# Sagittal Parameters and Clinical Outcomes in Cervical Spondylitis: The Cohort Analysis

**DOI:** 10.3390/diseases13020049

**Published:** 2025-02-06

**Authors:** Denis Naumov, Sergey Tkach, Natalia Linkova, Dmitrii Medvedev, Alexander Krasichkov, Olga Sokolova, Victoria Polyakova, Giuseppe Gullo, Piotr Yablonskiy

**Affiliations:** 1 Scientific Research Laboratory for the Development of drug Delivery Systems, St. Petersburg Research Institute of Phthisiopulmonology, 2-4 Ligovskii Prospect, St. Petersburg 191036, Russiaop.sokolova@spbniif.ru (O.S.);; 2 The Laboratory If the Fundamental and Translational Investigations of Aging, Institute of Experimental Medicine, Acad. Pavlov Street, 12, St. Petersburg 197022, Russia; 3 The Laboratory “Problems of Aging”, Belgorod National Research University, Belgorod 308015, Russia; 4 The Department of Social Rehabilitation and Occupational Therapy, St. Petersburg Medical and Social Institute, Kondratievsky St., 72A, St. Petersburg 195271, Russia; 5 Department of Radio Engineering Systems, Electrotechnical University “LETI”, 5F Prof. Popova Street, St. Petersburg 197022, Russia; 6 Obstetrics and Gynecology Unit, Villa Sofia Cervello Hospital, I.V.F. Public Center, University of Palermo, Via Trabucco, 180, 90146 Palermo, Italy; gullogiuseppe@libero.it; 7 Department of Hospital Surgery, Faculty of Medicine, St. Petersburg University, 7-9 Universitetskaya Ave., St. Petersburg 199034, Russia

**Keywords:** cervical spondylitis, predictors of complications, vertebrogenic pain syndrome, sagittal parameters

## Abstract

Background. Cervical spondylitis is accompanied by segmental instability and sagittal imbalance. The purpose of this work is to conduct a search of correlation between sagittal parameters and clinical outcomes in cervical spondylitis. Materials and Methods. The monocentric cohort study encompassed the clinical and radiological data of 59 patients who underwent reconstructive surgeries on the suboccipital, subaxial, and cervicothoracic spine. We evaluated local cervical sagittal parameters: cervical sagittal vertical axis (CSVA), T1 slope (T1S), **Health-Related Quality of Life**—HRQOL (Oswestry Disability Index—ODI)—and others pre- and postoperatively. Results. The duration of the therapeutic pause and T1S correlated with HRQOL. It revealed the direct relationship between the age of the patient and the value of CSVA. A significant predictor of postoperative complications is the level of comorbidity with an index of 7 or more on the Charlson scale. Conclusions. The factors influencing HRQOL in this pathology are the duration of the therapeutic pause and the magnitude of T1S compensation. Anterior reconstruction of the cervical spine in the presence of spondylitis yields a correction of the sagittal balance parameters. The leading predictors of complications from the surgical treatment of cervical spondylitis are the Charlson comorbidity index and the variant of anterior reconstruction.

## 1. Introduction

Infectious spondylitis is a heterogeneous group of diseases manifested by vertebral destruction, instability, soft tissue component formation, and pain syndrome [1,2,3,4]. The incidence of infectious spondylitis is estimated to range from 0.2 to 2.4 cases per 100,000 population annually [5].

Infectious spondylitis can develop as a result of the presence of four main factors. 1. Bacterial infections in other parts of the body (urinary tract infections, pneumonia, etc.), which can spread in the blood and affect the spine. 2. Spinal surgery that increases the risk of infection. 3. Spinal injuries that create pathways for bacteria to enter the spine. 4. Weak immune response, which makes organism more vulnerable to infections. Neurological symptoms are most typical for spondylitis of the cervical or thoracic spine. In typical cases, the pain is localized in the area of the intervertebral disk involved in the process and increases with physical exertion or percussion in the affected area. The pain can radiate to the abdomen, various parts of the leg, groin, and perineum.

The key criterion affecting the quality of life of patients with infectious cervical spondylitis is sagittal balance, whose restoration is one of the main goals of surgical treatment [6]. The standout parameters of sagittal balance in the cervical spine are CSVA (cervical sagittal vertical axis), T1S (Th1 slope), NTA (neck tilt angle), cLordo (cervical lordosis), and cKyph (cervical kyphosis) [7,8].

The strategy for the surgical treatment of cervical spondylitis is determined by the prevalence of bone destruction and the clinical features of disease [9,10]. The best clinical results in suboccipital spondylitis (Os-C2) are obtained by occipito-spondylodesis with transoral debridement and decompression [11,12,13], whereas in subaxial (C3-C7) lesions, isolated anterior reconstruction, i.e., corpectomy and spondylodesis, is effective [14,15,16].

The results of analyzing the literature revealed the absence of systematized data on the influence of cervical sagittal balance parameters in chronic infectious spondylitis on patients’ quality of life and the long-term results of surgical interventions. The purpose of this work is to conduct a search of correlation between sagittal parameters and clinical outcomes in cervical spondylitis.

## 2. Materials and Methods

The monocentric continuous cohort study was approved by the local ethical committee of the St. Petersburg Research Institute of Phthisiopulmonology (Meeting Minutes No. 417, dated 24 October 2023). The study adheres to the norms of the Declaration of Helsinki. The study materials were compiled retrospectively according to the following inclusion criteria: bacteriologically or histologically verified infectious cervical spondylitis; surgical treatment at the Scientific and Clinical Center for Spine Pathology at the St. Petersburg Research Institute of Phthisiopulmonology from 1 January 2017 to 31 December 2021; chronic nature of the infectious process (period from the appearance of the first symptoms to the moment of surgery not less than 3 months in the absence of the effect of complex conservative antibacterial therapy, carried out on tuberculous lesions according to the recommended regimes [17], in case of a nonspecific infectious process—not less than 2 months); patients over 18 years of age. The average age of the patients participating in the study was 51 ± 13 years. Exclusion criteria: discitis and spondylodiscitis corresponding to types A1-4 and B1-2 [18]; surgical treatment within the scope of isolated cervical epidural abscess drainage (true primary epiduritis) or diagnostic open/closed biopsy; initial level of neurological disorders corresponding to Frankel types A and B [18], due to the impossibility of performing the radiological examination of patients in the standing position; surgeries previously performed on the cervical spine.

In all cases of spondylitis, before the operation, X-ray-guided biopsy was performed for etiology verification (complex of laboratory methods used, including real-time polymerase chain reaction for M. tuberculosis DNA detection, bacteriological and morphological tests). After microorganism detection, drug-resistance testing was performed in all cases for target antibacterial therapy prescription. The duration of therapy was 8 weeks before and after surgery for pyogenic spondylitis and 12 months for TB spondylitis.

According to etiological verification, the clinical cohort included 32 patients with pyogenic and 27 patients with tuberculosis spondylitis. There was no difference in age, gender, localization of spondylitis, number of destructive vertebral bodies, neurological status and sagittal parameters before operation. Patients were comparable in their comorbidity status. General characteristics of the clinical cohort are presented in Table 1.

**Figure 1:**
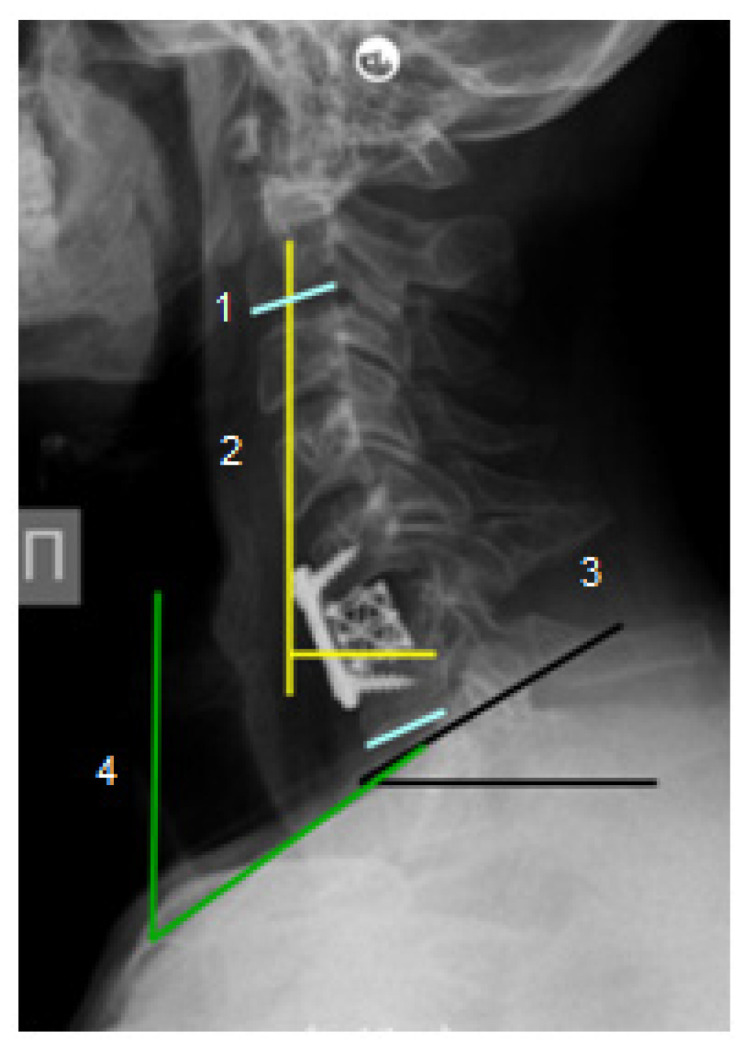
Cervical spine radiograph in the lateral projection in a patient with infectious cervical spondylitis. Symbols: 1—cervical lordosis C2-C7; 2—cervical sagittal vertical axis (CSVA); 3—Th1 slope (T1S); 4—neck tilt angle (NTA).

Neurological status was studied according to the Frankel scale [19] before surgery and at the time of hospital discharge. Follow-up was at 38 ± 4 months (mean: 3 years, 3 months). All patients underwent preoperative radiological examination, including cervical spine radiography in the lateral projection, computed tomography to determine the extent of bone destruction, and magnetic resonance imaging to assess intramedullary changes and the presence of epidural, para- and prevertebral abscesses. Sagittal balance parameters were evaluated by radiography: CSVA with the delineation of reference values for sagittal imbalance >4 cm, Th1 slope (>25°) and NTA (range limits 13° to 25°) [20]. Calculations were performed using Surgimap v2.3.2.1 software (Methuen, MA, USA) with the preliminary anonymization of data (Figure 1).

Simultaneous reconstructive surgeries were performed in all patients 3 months after the start of conservative antibacterial therapy (according to bacteriological results of X-ray guided biopsy). The surgical treatment strategy of suboccipital (Oc-C2) spondylitis included performing occipitospondylodesis (occipital plate and cervical screw fixation), transoral debridement (resection of anterior arch of C1 and dens of C2), and anterior spinal canal decompression. In the case of the subaxial (C2-C7) spondylitis, an isolated anterior reconstruction was performed. The surgical technique included an anterior right-sided Smith–Robinson approach follow by medial border of sternocleidomastoideus muscle. The anterior column was dissected sharply, resection of anterior longitudinal ligament was performed in standard manner, then the pathological vertebral bodies and pathological tissue was resected with a high-speed drill and Kerrison and pituitary rongeurs. Anterior decompression of the spinal canal included posterior longitudinal ligament resection and debridement of epidural abscess. Anterior fusion was performed using a titanium mesh cage with auto-bone graft from iliac crest for monosegmental spondylitis and a titanium mesh cage with auto-bone graft from iliac crest plus anterior locking plate for multisegmental (two or more vertebral motion segments) spondylitis. In two cases, posterior transpedicular fixation, posterior decompression, and debridement of the epidural abscess were performed. For anterior column distraction and local kyphosis correction we used the Caspar distractor with a transcorporal pin inserted in the central area of upper and lower vertebral bodies.

Reconstructions of the cervical-thoracic (C7-Th2) spine were performed from a combined surgical approach due to high risk of local kyphosis progression with-out posterior screw fixation and fusion. The first stage was the anterior (ventral) reconstruction, performed with standard surgical technique, followed by posterior instrumental screw fixation and fusion by auto-bone graft. At the same time, the isolated posterior fixation of the cervical spine due to the chronic nature of spondylitis and the presence of a pronounced destructive process on the part of the anterior column of the spine was performed by us only in two clinical observations.

The severity of vertebrogenic pain syndrome was assessed in 1–10 points by the Visual Analog Scale (VAS). VAS is a popular tool for the measurement of pain. VAS consists of asking the patient to mark a point on an ungraded 10-centimetre-long line that corresponds to the severity of pain. The left border of the line corresponds to the definition of “no pain at all”, the right one is “the most intense pain imaginable”.

The following parameters were recorded as analyzed parameters: duration of surgery in minutes and operative blood loss in mL; variant of anterior stabilization in 180° reconstructions (titanium mesh cage with autograft or titanium mesh cage with autograft combined with anterior locking plate); timing and nature of complications; quality of life of patients before and after reconstructive surgery using the ODI (Oswestry disability index) and NDI (neck disability index) scales. The ODI is one of the principal condition-specific outcome measures used in the management of spinal disorders. The ODI is used in the pain management of spinal disorders to evaluate the impact of the patient’s condition on their ability to perform daily lifestyle activities. The NDI is the most widely used and most strongly validated instrument for assessing self-rated disability in patients with neck pain. It has been used effectively in clinical and research settings in the treatment of various diseases.

We evaluated 6 predictors of postoperative complications. These were the Charlson comorbidity index (CCI), extent of bone destruction, variant of anterior spinal stabilization, etiology of spondylitis, gender, age of patients, and duration of the therapeutic pause (the duration from the appearance of the first symptoms of the disease to the treatment). The Charlson Comorbidity Index (CCI) was designed to predict long-term mortality, with regard to its reliability, concurrent validity, sensitivity, incremental, and predictive validity. The CCI is clinically useful not only to provide a valid assessment of the patient’s unique clinical situation, but also to demarcate major diagnostic and prognostic differences among subgroups of patients sharing the same medical diagnosis. The use of the CCI as one of the potential predictors of the development of postoperative complications is due to its universality, the completeness of the assessment of concomitant pathology, and a high level of prognostic significance, with an expected follow-up period of 10 years or more. At the same time, assessment of the somatic status of patients with infectious spondylitis is necessary due to the high incidence of severe concomitant pathology—acquired immunodeficiency syndrome, chronic viral hepatitis B, diabetes mellitus.

Statistical analysis of the data was performed using the Statistical Package for the Social Sciences program (SPSS), version 22.0 (SPSS Inc., Chicago, IL, USA). The tested quantitative parameters were checked for normality of distribution using the Kolmogorov–Smirnov and Shapiro–Wilk criteria. For all quantitative parameters, the level of two-sided significance was *p* < 0.05, indicating the non-normality of their distribution; therefore, the results are presented as M ± m and Me (min, max). The Mann–Whitney U criterion was used to assess the significance of differences in the duration of surgery and blood loss depending on the variant of anterior stabilization. The significance of the influence of sagittal balance parameters, the extent of bone destruction and the duration of the therapeutic pause on the severity of pain syndrome and the quality of life of patients was assessed using the Spearman correlation coefficient. The MANOVA test was used to assess the impact of the analyzing parameters group on patients’ quality of life, including a modified ODI score with the following levels of disability: 0–20 minimal disability; 21–40 moderate disability; 41–60 severe disability; 61–80 cripple, pain impinges on all aspects of patient’s life; and 81–100 patients are bed-bound. NDI scales with following levels were also used: 0–4 minimal disability; 5–14 mild disability; 15–24 moderate disability; 25–34 severe disability; more than 35—total disability. The significance level for MANOVA was 0,05, confidence intervals were 95%. For significance parameters, the Fisher LSD (least significant difference) post hoc test was used. The influence of qualitative parameters on the development of postoperative complications was tested by Pearson’s x^2^ criterion. Differences were recognized as statistically significant at the two-sided significance level *p* < 0.05.

## 3. Results

The study cohort consisted of 59 patients aged 26 to 75 years (M ± m—51 ± 13 years, Me—49 years) who underwent 63 consecutive reconstructive surgeries on the suboccipital (n_1_ = 16), subaxial (n_2_ = 39), and cervicothoracic (n_3_ = 8) spine. The duration of the therapeutic pause ranged from 3 to 21 months (M ± m—7 ± 5 months, Me—5 months). Pulmonary tuberculosis (8 cases), HIV infection (7 cases), multiple foci of bone destruction (6 cases), persistent urological infection (4 cases) and a historical incidence of sepsis (2 cases) were revealed in the comorbidity structure. The comorbidity index, according to the Charlson ME scale (1987), was M ± m—3.6 ± 2.2 points (min 1, max 7; Me 3 points). The patients’ ODI quality of life scores were 62.4 ± 11.5 preoperatively and 21.1 ± 4.7 postoperatively (*p* = 0.015). The dynamic of NDI scores was 33.4 ± 7.6 preoperatively and 18.4 ± 3.6 postoperatively (*p* = 0.036). The effect of the duration of the therapeutic pause on the patients’ quality of life was revealed to be significant. The shorter the therapeutic pause, the higher the maladaptation scores on the ODI and NDI scales (*r* = −0.436, *p* = 0.043). There was a direct correlation between the age of the patients and CSVA value. The older the patients, the greater its deviation (*r* = 0.528, *p* = 0.035). This can be explained by the age-related decrease in mobility and compensatory capabilities of the vertebral-motor segments; in particular, less compensation of the cervical lordosis due to an increase in T1S. The Charlson comorbidity level proved to be a significant predictor of the development of postoperative complications (*x*^2^ = 7.194, *p* = 0.027), whose highest number was noted in patients with an index score of 7 or more.

A statistically significant influence of Th1 vertebral tilt angle (T1S) on the severity of vertebrogenic pain syndrome and patients’ quality of life was revealed (*r* = 0.567, *p* = 0.022). However, CSVA (*r* = 0.176, *p* = 0.514) and NTA (*r* = −0.135, *p* = 0.617) did not influence these parameters. No significant effect of the number of destroyed vertebral-motor segments was found either on patients’ quality of life (*r* = −0.036, *p* = 0.872) or on the degree of change in sagittal balance parameters—CSVA (*r* = 0.409, *p* = 0.116), *T1S* (*r* = −0.373, *p* = 0.154) and *NTA* (*r* = −0.157, *p* = 0.562).

A one-way MANOVA test confirmed the influence of T1S and the duration of the therapeutic pause on health-related quality of life; a statistically significant MANOVA effect was obtained, F (15, 81) = 2.374, *p* = 0.007. The series of post hoc Fisher’s LSD tests revealed that comparisons were statistically significant, *p* < 0.05.

The results of an intergroup comparison of the duration of surgery and operative blood loss in patients undergoing surgery within the scope of corpectomy, anterior spondylodesis with titanium mesh cage with autograft (ACCF), and corpectomy with anterior spondylodesis with titanium mesh cage with auto bone combined with anterior locking plate (ACCF + AP) are presented in Table 2.

**Figure 2:**
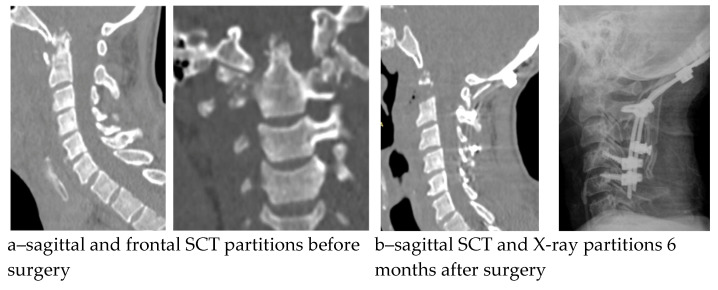
Pre- and postoperative radiological findings of a patient with C1-2 spondylitis.

**Figure 3:**
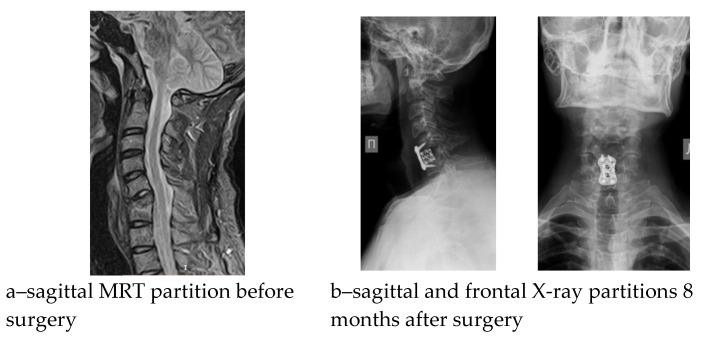
Pre- and postoperative radiological findings of a patient with C6-7 spondylitis.

Seven postoperative complications were noted in the cohort with a predominance in the AACF + AP group; however, the reconstruction variant was not proven to have a significant impact (*x*^2^ = 3.689, *p* = 0.297). In the early postoperative period, three patients had a pain syndrome of 5 to 6 VAS scores in the area of the autograft (rib autograft fragment), which was controlled by a course of non-steroidal anti-inflammatory drugs in combination with physiotherapeutic treatment. In one case, 3 months after the operation, the epidural abscess persisted: C4-5 hemilaminectomy, sanitation, and posterior instrumental fixation were performed. In three cases, instability of the spinal reconstruction zone was detected, manifested by the recurrence of vertebrogenic pain syndrome; in one case, it was detected against the backdrop of deep infection of the surgical intervention zone. The development period of the aforementioned complications was 6–8 months. Revision surgeries were performed within the scope of anterior respondylodesis with a titanium mesh cage with auto bone (n_1_ = 2) and anterior column debridement combined with posterior screw fixation (n_2_ = 1). Analysis of sagittal balance showed a significant change primarily in the T1S and NTA parameters, but no significant association of these changes with the etiology of spondylitis (Table 3). The results of the surgical treatment of suboccipital and subaxial chronic spondylitis are shown in Figure 2 and Figure 3.

## 4. Discussion

Treatment of infectious lesions of the cervical spine is based on three core principles: the need for etiologic verification, debridement of the focus of inflammation, and the correction of orthopedic complications. At the same time, cervical spondylitis is complicated early by secondary myelopathy with a high level of neurological deficit with a high complexity of invasive diagnostic manipulations, including X-ray or CT-guided biopsy, and “early” reconstructive interventions [20,21,22]. The publications focus on the surgical features of the treatment of this pathology, including surgical accesses, variants of fusion materials for anterior column restoration and regimes of antibacterial chemotherapy [23,24,25]. Only in recent years has the consideration of this disease begun to emphasize the assessment of patients’ quality of life, the possibility of minimizing surgical intervention, and the analysis of long-term surgical results from the perspective of local and global sagittal balance parameters [26,27,28]. The data obtained in this study are new from the point of view of predicting long-term results and determining the optimal method of surgical treatment aimed at restoring the parameters of sagittal balance, debridement of the inflammatory focus and improvement of the quality of life of patients. It has been shown for the first time that the restoration of sagittal balance parameters is a determining factor for improving the quality of life and reducing the risks of postoperative complications in patients with infectious lesions of the cervical spine.

Within the framework of this study, for the first time, a significant effect of T1S (Th1 slope) on the intensity of vertebrogenic pain syndrome and the quality of life of patients was established. It is associated with both changes in the biomechanics of the cervical and thoracic spine, in particular, an increase in local kyphosis and the formation of persistent musculotonic syndrome aimed at compensating for deformity. It should be noted that the effect of T1S on these parameters (vertebrogenic pain and quality of life) has been proven in patients with infectious spondylitis for the first time, and similar studies concerned only patients with post-traumatic deformities and Bechterew’s disease.

The consensus on surgical tactics for suboccipital infectious spondylitis is noteworthy [29]. For a long time, the priority direction of treatment of patients with infectious spondylitis was the rehabilitation and removal of destroyed vertebrae. However, the long-term results of these operations indicate a high frequency of secondary spinal deformities and a decrease in the quality of life of patients. The conducted research indicates that the combination of the sanitizing and reconstructive stages with the restoration of sagittal balance parameters provides the best long-term results. The high efficacy of occipitospondylodesis in combination with transoral debridement and anterior decompression has been described [26,27,30,31,32]. C1-C2 fusion (Harms-Magerl procedure, including C1 lateral mass screw fixation and C2 pars screw fixation) is recommended in the case of absence of neurological deficit and predominant lesion of the C2 dens with atlanto-axial segmental instability presentation [29]. In our cohort, we performed three atlantoaxial reconstructions within the scope of occipitospondylodesis and transoral C2 dens resection and spinal cord decompression due to the presence of different degrees of neurological complications.

Treatment strategy in subaxial spondylitis remains controversial. Some authors recommend supplementing anterior spondylodesis with a block plate, but the main argument in favor of this approach is the correction of sagittal balance parameters, primarily in multisegmental reconstructions. Surgical outcomes in monosegmental lesions remain comparable [24]. According to our data, the use of an additional cervical plating not only fails to guarantee a favorable outcome, but also increases the risks of postoperative complications, which may be associated with an increase in the duration of surgery and blood loss (risk factors for infections in the area of surgical intervention).

Among the limitations of this study, it is necessary to highlight the multicenter nature of the analyzed cohort. Other limitation of our investigation is the need to evaluate the results at a later date (10 years or more) from the point of view of changes in the parameters of the sagittal profile of the spine, and the dynamics of the state of adjacent vertebral motion segments, including degeneration of facet joints, intervertebral disks, as potential predictors of a decrease in the quality of life of patients.

## 5. Conclusions

Our study shows for the first time that the most frequent cause of such complications in the early postoperative period is infection in the area of surgical intervention, and in the late period—the formation of pseudarthrosis. The leading predictors of complications from the surgical treatment of cervical infectious spondylitis are the Charlson comorbidity index (7 points or more) and the variant of anterior reconstruction (use of a lockable cuff plate). Factors affecting the quality of life of patients with this pathology are the duration of the therapeutic pause and the value of T1S compensation. Anterior reconstructions of the cervical spine in infectious spondylitis allow us to correct the parameters of sagittal balance with the possibility of maintaining the achieved parameters over the long run. The improvement of the proposed treatment for infectious spondylitis of the cervical spine has practical significance to modern medicine.

## Figures and Tables

**Table 1:** Patients’ characteristics.

	TB * Spondylitis	Pyogenic Spondylitis	*p*
Age	50 ± 17 years, Me—48 years	52 ± 12 years, Me—50 years	>0.05
Gender	m: 15/f: 12	m: 17/f: 15	>0.05
Localization	Oc-C2: 9/C2-7: 18/C7-Th2: 3	Oc-C2: 7/C2-7: 21/C7-Th2: 5	>0.05
Number of VMS **	3.4 ± 0.2, Me—3	3.1 ± 0.5, Me—3	>0.05
Charlson index	3.1 ± 1.9	3.6 ± 2.3	>0.05
Sagittal parameters			
CSVA	3.9 ± 1.6	4.2 ± 2.2	>0.05
T1S	23.1 ± 15.2	27.4 ± 17.1	>0.05
NTA	41.9 ± 9.7	46.5 ± 6.1	>0.05
cKyph	32.6 ± 7.1	24.2 ± 5.9	>0.05

*—tuberculosis, **—vertebral motion segments.

**Table 2:** Intergroup comparative analysis of surgical performance in patients with infectious cervical spondylitis.

Operation Characterization	Duration of Operation, min (M ± m; Me)	Operative Blood Loss, mL (M ± m; Me)	Significance Level, *p*
ACCF	93 ± 24; 92	56 ± 17; 50	* *p* = 0.007 ** *p* = 0.020
ACCF + AP	120 ± 18; 115	104 ± 56; 100	

Assessment of the significance of differences was analyzed using the Mann–Whitney U criterion; *—significance for the duration of the operation; **—significance for blood loss. Note: ACCF—titanium block grating with autograft; ACCF + AP—auto bone combined with a bone block plate; M—average; m—average error; Me—median.

**Table 3:** Sagittal balance indices in patients with infectious cervical spondylitis.

Indicators Assessed	Before Surgery	After Surgery	*p* *
Tuberculous spondylitis			
CSVA	3.9 ± 1.6	3.4 ± 1.3	0.746
T1S	23.1 ± 15.2	16.3 ± 9.2	0.034
NTA	41.9 ± 9.7	24.8 ± 7.6	0.024
cKyph	32.6 ± 7.1	9.4 ± 5.6	0.007
Chronic nonspecific spondylitis			
CSVA	4.2 ± 2.2	3.7 ± 1.9	0.683
T1S	27.4 ± 17.1	19.3 ± 11.4	0.031
NTA	46.5 ± 6.1	27.2 ± 3.4	0.018
cKyph	24.2 ± 5.9	6.2 ± 3.1	0.009
Mean			
CSVA	4.1 ± 1.9	3.5 ± 1.4	0.657
T1S	26.1 ± 16.1	17.8 ± 10.3	0.038
NTA	44.8 ± 7.6	25.9 ± 5.2	0.016
cKyph	11.4 ± 6.2	4.1 ± 2	0.003

* Assessment of the significance of differences was analyzed using the Mann–Whitney U criterion.

## Data Availability

Data are contained within the article.
